# Supplementary figures and images for: Reprogrammed human lateral ganglionic eminence precursors generate striatal neurons and restore motor function in a rat model of Huntington’s disease
Source: Stem Cell Res Ther. 2024 Nov 22;15:448. doi: 10.1186/s13287-024-04057-9 (PMC11583420; doi:10.1186/s13287-024-04057-9)

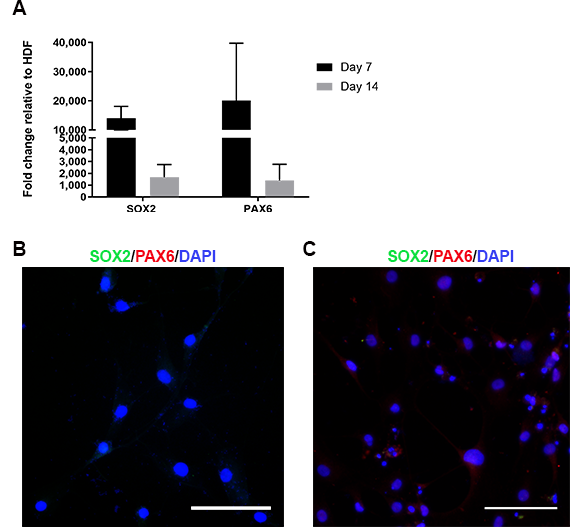

Supplement: Supplementary file 1 — Supplementary Material 1: Supplementary Fig. 1: SOX2 and PAX6 expression reduces over the course of reprogramming and is no longer present in differentiated hiLGEPs. (A) Graph demonstrating the gene expression of SOX2 and PAX6 at Day 7 and Day 14 of reprogramming. Data represent fold changes in mRNA expression relative to HDFs with mean ± SEM and n = 3 independent cell lines. SOX2 and PAX6 protein expression in (B) reprogrammed hiLGEPs and (C) at Day 14 of differentiation. Scale bars: 100 μm. [file 13287_2024_4057_MOESM1_ESM.tif]
